# Type 1 diabetes mellitus and risk of incident epilepsy: a population-based, open-cohort study

**DOI:** 10.1007/s00125-016-4142-x

**Published:** 2016-10-31

**Authors:** George E. Dafoulas, Konstantinos A. Toulis, Dougall Mccorry, Balachadran Kumarendran, G. Neil Thomas, Brian H. Willis, Krishna Gokhale, George Gkoutos, Parth Narendran, Krishnarajah Nirantharakumar

**Affiliations:** 1 Institute of Applied Health Research, University of Birmingham, Birmingham, UK; 2 First Department of Propaedeutic Internal Medicine, Diabetes Center, National and Kapodistrian University of Athens Medical School, Athens, Greece; 3 Department of Endocrinology, 424 General Army Training Hospital, Thessaloniki, Greece; 4 Department of Neuroscience, University Hospitals Birmingham NHS Foundation Trust, Birmingham, UK; 5 Centre for Computational Biology, University of Birmingham, Birmingham, UK; 6 Centre for Endocrinology, Diabetes and Metabolism, Institute of Metabolism and Systems Research, University of Birmingham, Room 229, Medical School, College of Medical and Dental Sciences, Edgbaston, Birmingham, B15 2TT UK; 7 University of Birmingham, Birmingham, UK

**Keywords:** Epilepsy, Hypoglycaemia, Insulin, Seizures, Type 1 diabetes mellitus

## Abstract

**Aims/Hypothesis:**

The aim of this research was to explore the relationship between incident epilepsy and type 1 diabetes in British participants.

**Methods:**

Using The Health Improvement Network database, we conducted a retrospective, open-cohort study. Patients who were newly diagnosed with type 1 diabetes mellitus at the age of ≤40 years were identified and followed-up from 1 January 1990 to 15 September 2015. These patients, identified as not suffering from epilepsy at the time of diagnosis, were randomly matched with up to four individuals without type 1 diabetes mellitus, based on age, sex and participating general practice. A Cox regression analysis was subsequently performed using Townsend deprivation index, cerebral palsy, head injury and learning disabilities as model covariates.

**Results:**

The study population consisted of a total of 24,610 individuals (4922 with type 1 diabetes and 19,688 controls). These individuals were followed up for a mean of 5.4 years (approximately 132,000 person-years of follow up). Patients with type 1 diabetes were significantly more likely to be diagnosed with epilepsy during the observation period compared with controls (crude HR [95% CI]: 3.02 [1.95, 4.69]). The incidence rate was estimated to be 132 and 44 per 100,000 person-years in patients and controls, respectively. This finding persisted after adjusting for model covariates (adjusted HR [95% CI]: 3.01 [1.93, 4.68]) and was also robust to sensitivity analysis, excluding adult-onset type 1 diabetes mellitus.

**Conclusions/Interpretation:**

Patients with type 1 diabetes are at approximately three-times greater risk of developing epilepsy compared with matched controls without type 1 diabetes. This should be considered when investigating seizure-related disorders in patients with type 1 diabetes mellitus.

## Introduction

Type 1 diabetes mellitus is characterised by the immune-mediated destruction of pancreatic beta cells leading, ultimately, to the loss of insulin production and lifelong dependence on exogenous insulin. The disease affects over 370,000 adults in the UK, approximately 10% of the adult diabetic population [1].

Comorbidities associate with type 1 diabetes, either due to a common etiopathogenesis (as in autoimmune polyglandular syndromes) or due to metabolic (e.g. acute ketosis or chronic vascular) and non-metabolic (e.g. depression) complications of the disease. The potential association between type 1 diabetes and neurological conditions, in particular epilepsy, has recently received intense scrutiny [2–4]. Epidemiological evidence suggests that the prevalence of type 1 diabetes in patients with idiopathic generalised epilepsies [5] and the prevalence of epilepsy-related disorders in patients with type 1 diabetes may both be increased [6]. Furthermore, a major acute metabolic complication of type 1 diabetes, diabetic ketoacidosis, was found to be more frequent in patients with pre-existing epilepsy [7]. More recently, a population-based study from Asia reported that the risk of developing epilepsy was approximately three times higher in patients with type 1 diabetes compared with controls [4].

On the other hand, the evidence outlined above has not been universally replicated. A large study in the Australian population reported no difference in the prevalence of epilepsy in type 1 diabetes [8]. However, the primary design of this study did not address important variables, such as the potential distorting effect of age (which was not controlled for in the study by Chou et al [4]) and the possibility of a bidirectional relationship between type 1 diabetes and epilepsy [9]. Furthermore, the potential contribution of genetic background to the association between type 1 diabetes and epilepsy warrants confirmation in different populations. Finally, the relative rarity of the outcome (epilepsy in patients with type 1 diabetes) and the possibility of type I error inflation in the study by Chou et al [4], necessitates the replication of these findings in a different cohort.

In this study, we aimed to explore the relationship between epilepsy and type 1 diabetes in a large UK population. We conducted a retrospective, open-cohort study using available patient data residing in The Health Improvement Network (THIN) database.

## Methods

### Selection of patients

The study population (‘exposed patients’) included all patients who were newly diagnosed with type 1 diabetes at the age of ≤40 years during the study period (study start: 1 January 1990; study end: 15 September 2015). Participants were selected based on the criteria of no diagnosis of epilepsy at the time of diagnosis with type 1 diabetes. Individuals were eligible for inclusion if they had been registered with their general practice for ≥ 12 months. Patients with type 1 diabetes were followed up from the index date until the first of the following events (exit date): (1) patient died; (2) patient left health practice; (3) last data collection from health practice; (4) patient diagnosed with epilepsy; (5) or patient reached 50 years of age. The latter criterion was included since beyond this age many other comorbidities may give rise to epilepsy.

A diagnosis of type 1 diabetes was defined as the presence of a specific medical (Read) code indicating type 1 diabetes and verified by the presence of treatment with insulin and the absence of treatment with any oral glucose-lowering agent other than metformin.

### Selection of controls

Individuals with type 1 diabetes were randomly matched with up to four control individuals who were registered at the same general practice. Control individuals did not have a diagnosis of type 1 diabetes or epilepsy. Age at index date (to within 1 year), sex and participating general practice were use as matching variables.

### Outcomes and covariates

The primary outcome of the study was the relative risk of incident diagnosis of epilepsy during the observation period, expressed as HR. The accuracy of the coding for epilepsy in the THIN database has been independently verified in a previous study [10]. Covariates, selected on the basis of biological plausibility, were used in the adjusted analyses; covariates included Townsend deprivation index, cerebral palsy, head injuries and learning disability. These covariates were not matched.

### Statistical analysis

Baseline comparisons in continuous and dichotomous/categorical characteristics were performed using the Student’s *t* test and χ^2^ tests, respectively. A proportional hazards model using Cox regression analysis was used for the calculation of crude HR and adjusted HR, offset by the person-years of exposure to determine whether the exposure to type 1 diabetes was associated with the risk of epilepsy. The proportional hazards assumption (PHA) was tested before Cox regression analysis using Schoenfield and scaled Schoenfield residuals. Point estimates of HRs were calculated with 95% CI and the statistical significance threshold was set at 0.05. To explore whether any association was different in adults and children, a sensitivity analysis was performed limiting to patients with a diagnosis of type 1 diabetes (and their respective controls) at the age of 18 years or less. All analyses were performed using Stata/MP (StataCorp, 2015, Stata Statistical Software: Release 14 [version 14.0], College Station, TX, USA). The study was approved by the relevant Scientific Review Committee (SRC; reference number 16THIN068).

## Results

### Cohort characteristics

The study population consisted of a total of 24,610 individuals (14,530 male and 10,080 female). The patients (4922 exposed to type 1 diabetes and 19,688 controls) were followed up for a mean of 5.4 years (totalling approximately 132,000 person-years of follow up). The mean age of each cohort was 17.9 years (Table 1). In total, 81 incident cases of epilepsy were recorded during the observation period (Table 2). A diagnosis of learning disability and cerebral palsy were recorded in 85 individuals (0.3% of the total population) and 27 individuals (0.1% of the total population), respectively. Approximately 8.6% of the total population (*n* = 2125) had a previous diagnosis of head injury, including skull fractures. Table 1 provides a summary of the key study characteristics.

**Table 1:** Baseline characteristics of the study population

Characteristics	Exposed to type 1 diabetes	Control	*p* value
	*n* = 4922	*n* = 19,688	
Age, years	17.9 (10.7)	17.9 (10.7)	0.97
Male, *n* (%)	2906 (59.0)	11,624 (59.0)	0.99
Follow up, years	5.38 (4.0)	5.36 (4.0)	0.75
Townsend deprivation index, *n* (%)			0.49^b^
1	1080 (22.0)	4429 (22.5)	
2	949 (19.3)	3814 (19.4)	
3	1027 (20.9)	3932 (20.0)	
4	874 (17.8)	3645 (18.5)	
5	723 (14.7)	2772 (14.1)	
NA	269 (5.5)	1096 (5.6)	
Cerebral palsy, *n* (%)	3 (0.1)	24 (0.1)	0.28
Head injury^a^, *n* (%)	424 (8.6)	1701 (8.6)	0.95
Learning disability, *n* (%)	24 (0.5)	61 (0.3)	0.06

Data are presented as mean (SD) or *n* (%).

^a^Includes skull fracture

^b^ *p* value derived from categorical Townsend deprivation index data in the exposed vs control cohorts

NA, not available

*p* values are derived from Student’s *t* tests or χ^2^ test

**Table 2:** Risk of epilepsy in patients with type 1 diabetes mellitus in UK

Variable	Exposed to type 1 diabetes	Controls	Exposed to type 1 diabetes at ≤18 years of age^a^	Controls^a^
*n*	4922	19,688	2893	11,550
Person-years, years	26,499	105,598	16,015	62,525
Incident diagnosis of epilepsy, *n*	35	46	24	28
Incidence rate (per 100,000 person-years), *n*	132	44	150	45
Mean follow up, years	5.52	5.42	5.54	5.41
Unadjusted HR (95% CI)	3.02 (1.95, 4.69)^***^		3.44 (1.99, 5.96)^***^	
Adjusted HR (95% CI)^b^	3.01 (1.93, 4.68)^***^		3.40 (1.97, 5.88)^***^	

^a^Limited to individuals with onset of type 1 diabetes at ≤18 years of age and age-matched controls

^b^Adjusted for social deprivation, cerebral palsy, head injury and learning disability

***p < 0.001, individuals with type 1 diabetes vs controls

### Risk of incident epilepsy

Patients with type 1 diabetes were significantly more likely to be diagnosed with epilepsy during the observation period compared with controls (crude HR [95% CI]: 3.02 [1.95, 4.69]; *p* < 0.001; Table 2). The incidence rate was estimated at 132 and 44 per 100,000 person-years in type 1 diabetic patients and controls, respectively. This finding remained robust after adjusting for key covariates (adjusted HR [95% CI]: 3.01 [1.93, 4.68], Table 2).

### Sensitivity analysis

In patients diagnosed with type 1 diabetes at the age of 18 years or less, the risk of developing epilepsy during the observation period was significantly higher compared with controls (crude HR [95% CI]: 3.44 [1.99, 5.96], Table 2). Again, these findings remained unchanged following adjustments for key covariates (adjusted HR [95% CI]: 3.40 [1.97, 5.88], Table 2).

## Discussion

In this population-based, open cohort study, patients with type 1 diabetes were found to be at approximately three-times greater risk of developing epilepsy than matched controls without type 1 diabetes. This increase in the risk of epilepsy was robust to sensitivity analyses, including subgroups of adolescent patients with type 1 diabetes.

The validity this study confers to the literature is important in several aspects. First, this study included the largest cohort of patients with newly diagnosed type 1 diabetes (*n* = 4922 patients) to date. Furthermore, the temporal sequence (diagnosis of type 1 diabetes followed by a diagnosis of epilepsy) was ascertained by a reproducible, computerised procedure. Importantly, key potential modifiers of the outcomes, such as age [9], learning disabilities and related comorbidities were taken into account in the analysis, either as a matching variable (age) or as model covariates. Finally, appropriate sensitivity analyses were undertaken to explore whether any relationship identified was age dependent (adult vs adolescent type 1 diabetes).

The findings of the present study are directly comparable with the recent report by Chou et al that explored the association of type 1 diabetes with incident epilepsy in an Asian population [4]. Compared with our study, there is a potential for differences in ethnic and genetic backgrounds of participants, a notable disparity in the epilepsy incidence rate (which was approximately three times greater in the Asian cohort compared with our study), and differences in study size and analysis (age was not used as a matching variable and there were fewer type 1 diabetic patients in the Asian cohort). Nonetheless, the findings from the research by Chou et al were practically identical to those in the present study [4]. The consistently reported three-time greater risk of incident epilepsy in type 1 diabetes patients, independently validated in, at least, the cohort in the present study and that in the study by Chou et al, suggests a finding of relevance rather than artefact. On the other hand, it should be noted that patients with type 1 diabetes are closely monitored in the healthcare system and this might, in itself, increase the chance of being diagnosed with other conditions, such as epilepsy.

The mechanism of association between type 1 diabetes and epilepsy remains unclear. It may relate to shared genetic or shared autoimmune factors [9, 11, 12], or the result of cerebrovascular damage resulting from metabolic abnormalities [13]. Clarification of the underlying mechanism would possibly facilitate the recognition of those at risk, help to manage and reduce this risk, as well as accelerate necessary changes in patient care.

Whilst the association between type 1 diabetes mellitus and epilepsy would ideally be validated in a prospective setting, the results of our study suggest that it is advisable for healthcare professionals to seriously consider epilepsy, alongside hypoglycaemia, in the differential diagnosis of seizure-related disorders in patients with type 1 diabetes.
